# Associations of bulk tank milk free fatty acid concentration with farm type and time of year

**DOI:** 10.3168/jdsc.2024-0667

**Published:** 2024-12-16

**Authors:** Hannah M. Woodhouse, Stephen J. LeBlanc, Trevor J. DeVries, Karen J. Hand, David F. Kelton

**Affiliations:** 1 Department of Population Medicine, University of Guelph, Guelph, ON, Canada N1G2W1; 2 Department of Animal Biosciences, University of Guelph, Guelph, ON, Canada N1G2W1; 3 Precision Strategic Solutions, Puslinch, ON, Canada N0B2J0

## Abstract

Triacylglycerol (TAG) hydrolysis yields free fatty acids (FFA) and ≥1.20 mmol FFA/100 g of milk fat in bulk tank milk is associated with off-flavor, rancidity, reduced foam stability, and inhibited cheese coagulation. The objective of this study was to compare milk FFA concentrations among conventional (CON), organic (ORG), and certified grass-fed (CGF) dairy farm types in Ontario (ON), Canada, and describe monthly and yearly FFA patterns. Bulk tank FFA data measured at every milk pick-up from August 2018 to December 2022 were collected from all dairy farms in ON and averaged by month. A mixed model of monthly average FFA with herd as a random effect was used to investigate associations with month, year, and farm type. There were 171,843 observations from 3,771 farms over 53 mo (2 mo were excluded due to FFA calibration concerns). Ninety-seven percent (n = 166,355) of observations were from CON farms (n = 3,659), and the other 3% (n = 5,488) were from ORG (n = 72) and CGF (n = 40) herds. Conventional farms had the lowest overall average FFA (0.83 mmol/100 g of fat) with 7% (n = 11,645) of monthly averages ≥1.20 mmol/100 g of milk fat. Grass-fed herds had the highest overall average FFA (1.10 mmol/100 g of fat), and 23% (n = 842) of months had elevated FFA averages. Seventy-five percent (n = 30) of CGF farms had at least 1 elevated monthly average FFA over the 53 mo. In the mixed model, monthly average FFA levels were lower in May (*β* = −0.02 to −0.21) and higher in July (*β* = 0.01 to 0.12) than in other months. Conventional herds had lower monthly average FFA than CGF herds (*β* = −0.27, 95% CI [−0.18, −0.35]) or ORG herds (*β* = −0.08, 95% CI [−0.01, −0.14]). This research suggests that bulk tank milk FFA concentration varies among farm types, months, and years. The mechanisms underlying these associations warrant further investigation.

## Highlights

• FFA impair milk quality, and limited data are available in North America.
• Bulk tank FFA data on all Ontario, Canada, dairy farms were collected for this study.
• Data were analyzed by farm type, month, and year.
• Average monthly FFA differed by farm type, and conventional herds were lowest.
• Highest FFA were observed in grass-fed herds, July and September, and 2018.

## Graphical Abstract

**Figure:**
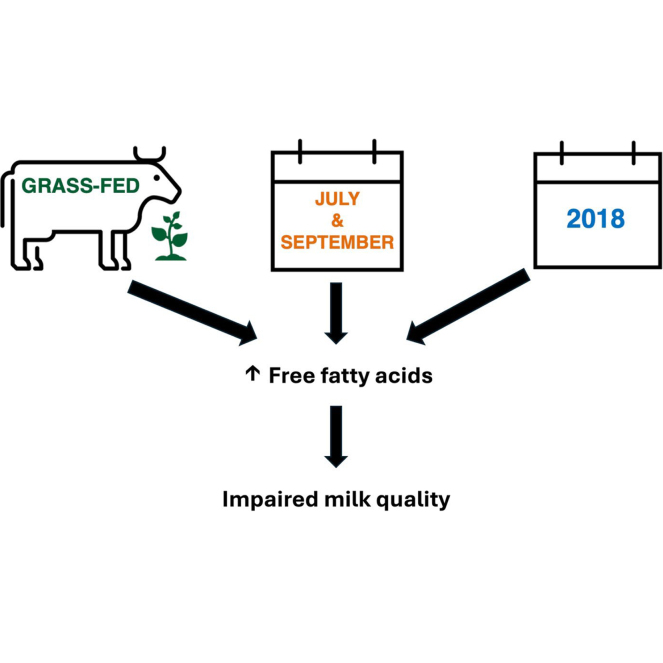
**Summary:** Elevated levels of free fatty acids (FFA) in milk (≥1.2 mmol/100 g of fat) impair milk quality. Many factors influence FFA levels, and this research aimed to identify farm type, monthly, and yearly factors and describe FFA patterns in Ontario, Canada. Average monthly FFA concentrations from all Ontario dairy farms from August 2018 to December 2022 were included in the dataset, and a mixed model was performed. The monthly average FFA level across all farm types was 0.83 mmol/100 g of fat, with 7% of monthly FFA averages ≥1.2 mmol/100 g of fat. Grass-fed herds, July and September, and 2018 were associated with higher FFA levels than other farm types, months, and years. Further research is required to identify the mechanisms underlying these associations.

Free fatty acids (**FFA**) are products of milk fat breakdown (Deeth, 2006; Hantsis-Zacharov and Halpern, 2007). Disruption of the milk fat globule membrane exposes triacylglycerol (**TAG**) molecules to lipolytic enzymes that cleave TAG and release fatty acids, making them “free” (Deeth, 2006; Hantsis-Zacharov and Halpern, 2007). Free fatty acids exist naturally in milk but should represent less than 0.1% of milk fat in whole raw milk (Lindmark Månsson, 2008). Around concentrations ≥1.20 mmol FFA/100 g of fat (Wiking et al., 2017), there may be issues with milk nonfoaming, rancidity, reduced shelf life, or cheese coagulation (Deeth, 2006; Skeie, 2007; Kamath et al., 2008). Therefore, elevated FFA poses a threat to the satisfaction of dairy processors and consumers.

Bulk tank FFA quantification in Canada began on Ontario (**ON**) dairy farms at every pick-up in August 2018. The motive for testing was to investigate industry FFA levels in response to processor and consumer milk quality concerns (some reports of problems with milk foam formation for specialty coffees) and to investigate contributing factors. There is currently no upper regulatory limit for FFA or penalty for farms with elevated FFA in ON, Canada.

The Canadian dairy operates under a quota-based supply-managed system to meet domestic consumer demand (CDC, 2020). Each producer has a daily quota allowance in kilograms of butterfat that keeps milk production relatively consistent throughout the year (CDC, 2020). During the lead-up to holidays (such as Thanksgiving and Christmas), milk demand typically increases, and each province's milk marketing board may respond by adding incentive days that allow for increased milk production in the corresponding month (CDC, 2020). Producers respond to incentives through herd management strategies, such as additional fat supplements in the ration or extending lactation length before dry-off, both of which may increase FFA (de Koning et al., 2003; Rasmussen et al., 2006). Incentive days can also occur at other times of the year during increased demand in milk markets, but this can change from year to year. Therefore, incentive days inconsistently align with certain months.

Most (97%) of herds in ON are milked conventionally (DFO, 2022). Canadian organic (**ORG**) and certified grass-fed (**CGF**) farms have increased forage (such as pasture, dry hay, and grass silage) requirements that could affect the milk fatty acid profile by increasing the ratio of n-3 to n-6, especially in CGF herds (Farhang, 2020). This is because ORG herds must have 60% of their DMI from forages throughout the year, whereas 75% of CGF herd diets must come from forages (Farhang, 2020; Mongeon, 2021). The composition of ORG and CGF cow diets can change depending on what forages are available (Farhang, 2020), and frequent alterations to the lactating cow ration may influence FFA levels (Hanuš et al., 2008). In addition, grass silage forages are more likely to contain greater amounts of butyric acid, which may risk increased FFA (Ciani et al., 2008; Schwendel et al., 2015a). However, the use of pasture (high in n-3 UFA) in the diets of ORG and CGF herds and the prohibition of fat supplementation (high in SFA) could reduce FFA (Wiking et al., 2003; Thomson et al., 2005; Schwendel et al., 2015b). Therefore, divergent risk factors exist for elevated FFA in ORG and CGF herds compared with conventional (**CON**) herds.

In studies conducted in New Zealand and Europe (Evers and Palfreyman, 2001; Wiking et al., 2019; Priyashantha et al., 2021), researchers have reported seasonal relationships with FFA. Lower FFA levels were reported for these countries in the spring and summer (Evers and Palfreyman, 2001; Wiking et al., 2019; Priyashantha et al., 2021). However, this could be confounded by seasonal calving and greater use of pasture for all farm types than in Canada (de Koning et al., 2003; Schwendel et al., 2015b). In ON, Canada, other aspects of milk quality, such as SCC and bacteria count, have been influenced by season (Perkins et al., 2009; Shock et al., 2015), but the relationship between time of year and FFA levels has not been investigated. Our objective was to compare bulk tank milk FFA concentrations among CON, ORG, and CGF farm types in ON and describe monthly and yearly FFA patterns. We hypothesized that FFA would be greater in CON herds and in the summer and fall months.

A retrospective observational cohort study was conducted. All licensed milk bulk tanks on dairy farms that had a milk pick-up between August 2018 and December 2022 (n = 3,771) were included in this analysis. Bulk tank milk FFA concentration and farm type data (CON, ORG, or CGF) from August 2018 to December 2022 were obtained from Dairy Farmers of Ontario (DFO), the single cooperative marketing board for dairy cattle farms in ON, and matched to the pick-up date. Herds that were both ORG and CGF (n = 8) were omitted from the analyses.

Before transfer from the bulk tank to the transport truck, milk was agitated in the bulk tank for at least 2 min, and then a 50-mL sample was taken by the milk truck driver with a stainless-steel dipper through the lid on the top of the bulk tank. Samples were not preserved but were kept at 4°C while being transported to the Guelph Food Laboratory (Guelph, ON, Canada). Samples were transported to a refrigerator within 8 h and to the laboratory within 2 d. At the laboratory, samples underwent FFA testing using FT-infrared spectroscopy with FOSS MilkoScan machines (Wiking et al., 2017). Free fatty acid concentrations were reported as mmol of total FFA/100 g of fat.

Monthly average FFA levels were calculated for each farm to smooth day-to-day variation. Upon inspection, the monthly average FFA values in February 2020 and April 2021 were markedly different from the other months and years, with a smaller variation, median, and mode. We suspected a calibration error, and although there was no way to confirm this, we excluded the data from these 2 mo from the analyses.

Using the standard statistical software STATA (version 16.0; StataCorp), data from CON, ORG, and CGF farms were analyzed and compared. Researchers were not blinded to any study data. Box plots were created to describe the distribution of average FFA by month and year and among farm types. We built a mixed model with monthly average FFA as the outcome, farm type (CON, ORG, or CGF), month, and year as fixed effects, and herd as a random effect. The referent categories for month, year, and farm type were selected based on the category with the lowest average FFA in the descriptive statistics. Correlation among the predictor variables was assessed using Spearman's rank correlation coefficient (r > |0.8|) to identify variables that were colinear, but none were present. Biologically plausible interactions were tested, but none had *P* < 0.05 to be considered in the model. For each variable, *β*-coefficients and 95% CI were reported, and a cut-off *P*-value <0.05 was used to determine whether to retain farm type, month, and year in the model. Repeated monthly average FFA measures of each farm were accounted for and included in the model, with the unstructured covariance structure chosen based on the lowest model Aikake's information criterion.

There were 171,843 monthly average FFA values from 3,771 bulk tanks. Ninety-seven percent (n = 166,355) of observations were from CON farms (n = 3,659), and the other 3% were from ORG (n = 72) and CGF (n = 40) herds. The overall monthly average FFA for all farms over the entire study period was 0.83 ± 0.33 mmol/100 g of fat.

Conventional herds had the lowest overall monthly average FFA (0.83 mmol/100 g of fat; minimum and maximum 0.04 and 7.68) with 7% (n = 11,645) of elevated monthly average FFA levels (≥1.20 mmol/100 g of fat), whereas CGF herds had the highest overall monthly average FFA (1.10 mmol/100 g of fat; 0.15 and 6.63) with 23% (n = 842) of elevated monthly average FFA samples. Organic herds had an overall monthly average FFA of 0.89 mmol/100 g of fat (0.31 and 7.09) with 12% (n = 414) of elevated monthly average FFA levels. Over 53 mo, 30% (n = 1,082) of CON herds had at least 1 elevated monthly average FFA concentration, whereas 55% (n = 40) of ORG and 75% (n = 30) of CGF herds had at least 1 elevated monthly average FFA.

The lowest monthly average FFA for all farm types was in May (Figure 1). The highest monthly average FFA for CON herds was in July and September. Grass-fed herds had a higher monthly FFA average in December (1.20 mmol/100 g of fat) than in other months. Organic herds had the most consistent FFA throughout the years (0.87–0.92 mmol/100 g of fat) compared with other farm types (Figure 2). Figure 3 illustrates FFA by month and year for CON, ORG, and CGF farms.

**Figure 1:**
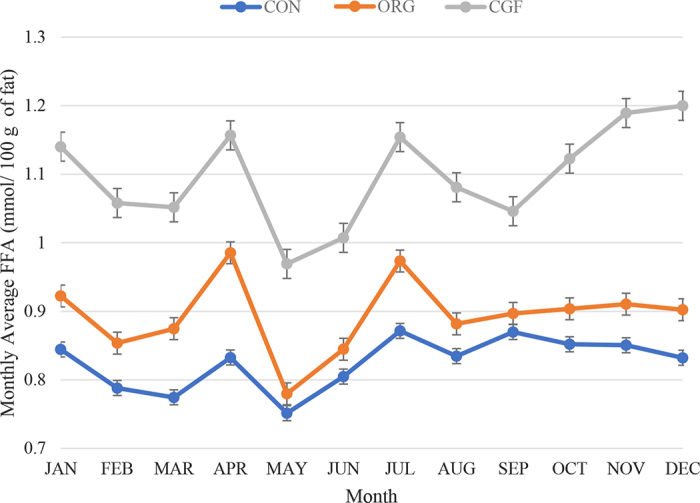
Monthly mean ± SE free fatty acid (FFA) concentration in bulk tank FFA by month for conventional (CON, n = 3,659), organic (ORG, n = 72), and certified grass-fed (CGF, n = 40) farms. The data are from all (n = 3,771) dairy farms in Ontario, Canada, from August 2018 to December 2022. There was a significant difference in monthly average FFA between farm types (*P* < 0.01).

**Figure 2:**
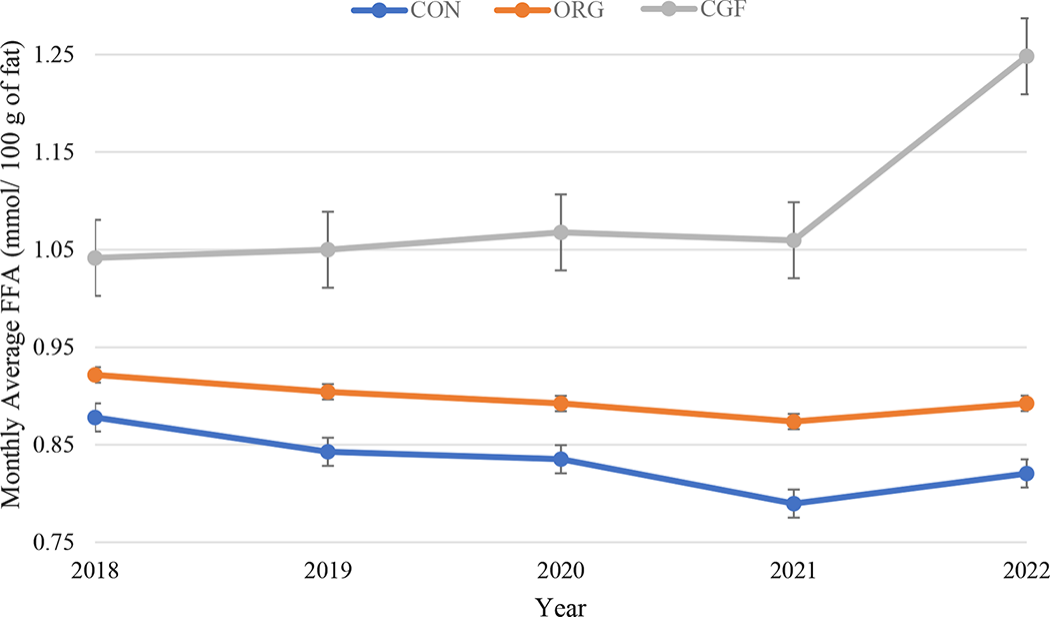
Annualized monthly average ± SE free fatty acid (FFA) concentration in bulk tank FFA for conventional (CON, n = 3,659), organic (ORG, n = 72), and certified grass-fed (CGF, n = 40) farms. The data are from all (n = 3,771) dairy farms in Ontario, Canada, from August 2018 to December 2022. There was a significant difference in monthly average FFA between farm types (*P* < 0.01).

**Figure 3:**
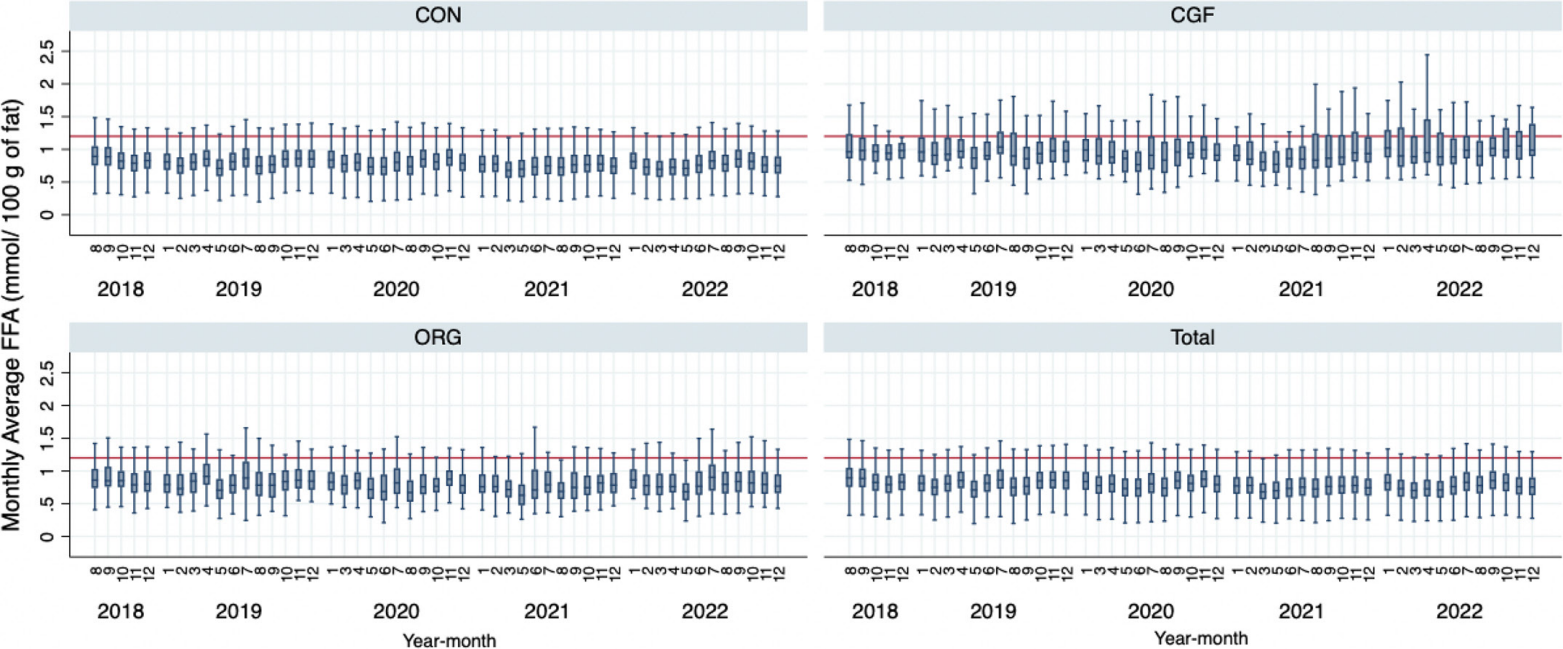
Box plots of monthly average free fatty acid (FFA) concentration (outside values not shown, 1 = January, 2 = February, and so on) for Ontario, Canada, conventional (CON) farms (n = 3,659), organic (ORG) farms (n = 72), certified grass-fed (CGF) farms (n = 40), and all farms (n = 3,771) from August 2018 to December 2022. The red horizontal line represents the threshold of 1.20 mmol FFA/100 g milk fat associated with an increased risk of milk quality concerns. Boxes represent the interquartile range, black lines inside the boxes represent the median value, and the whiskers represent the maximum and minimum values (excluding outliers). Due to calibration concerns, FFA values for February 2020 and April 2021 are not included.

Organic farms had the most stable monthly average FFA concentration across 4 yr, whereas CGF farms were the most variable (Figure 2). The average concentration of FFA was 0.19 mmol/100 g of fat greater in 2022 than in 2021 in CGF herds. Conventional herds had the highest monthly average FFA in 2018 and the lowest in 2021. Organic farms also had their lowest average FFA in 2021 and highest in 2018, but this was not the case in CGF herds, which had their lowest monthly average FFA in 2018.

Compared with CON herds, CGF herds had 0.27 mmol greater FFA/100 g of fat, and ORG herds had 0.08 mmol greater FFA/100 g of fat (*P* < 0.01). Both month and year were associated with monthly average FFA (*P* < 0.01). Monthly average FFA levels in May were 0.02 to 0.12 mmol/100 g of fat lower (*P* < 0.01) than other months. Monthly average FFA levels were greatest in July, with a 0.01 to 0.12 mmol/100 g of fat greater FFA concentration (*P* < 0.01) than other months. The intraclass correlation coefficient was 68%, indicating that most of the monthly average FFA variation comes from differences between farms (such as farm type) rather than the month or year.

Over 4 yr, we described bulk tank milk FFA patterns and identified differences among CON, ORG, and CGF dairy farms in ON, Canada. Monthly average FFA levels were lower in the spring, and late summer and fall (*P* < 0.01), which aligned with our hypothesis. Contrary to our expectation, CON herds had the lowest monthly average FFA concentration, and CGF herds had the highest (*P* < 0.01).

Grass-fed farms had the greatest monthly average FFA, and ORG farms had a higher monthly average FFA than CON herds. In the mixed model, CON herds had lower FFA than CGF or ORG herds. A study by Wiking et al. (2019) out of Denmark detected the opposite and reported lower FFA in ORG herds than in CON herds. However, they did not include CGF farms in the study. The lower FFA levels observed in ORG herds were suggested because pasture (a large part of ORG cow diets) is high in UFA, which may help produce smaller milk fat globules that have greater surface tension and stability (Wiking et al., 2003, 2019; Schwendel et al., 2015a). The differences in our study results may be attributable to factors such as shorter growing seasons in Canada due to more extreme winter and summer temperatures than some parts of Europe and New Zealand (Wiking et al., 2019; Farhang, 2020). Those shorter Canadian growing seasons could limit fresh pasture access and diversity for ORG and CGF herds, which means frequent feed changes and greater dependence on stored forages (Farhang, 2020).

Frequent feed changes (ie from pasture to stored forages depending on the growing season) may influence FFA levels (Hanuš et al., 2018). Ontario ORG and CGF herds alter their rations frequently to maintain the required forage levels (Farhang, 2020; Mongeon, 2021). Grass-fed herds had a higher monthly average FFA (*P* < 0.01) in 2022 compared with the previous years, and that concentration exceeded the FFA sensory threshold of 1.20 mmol FFA/100 g of fat. A warmer, dryer, and shorter growing season in 2022 compared with 2021 could explain these yearly differences (Government of Canada, 2022). This is because these farms may have relied on stored forages to supplement pasture, even during the summer months. Stored forages can contain high levels of butyric acid, which is a short-chain SFA that can easily be broken down into FFA (Ciani et al., 2008; Lindmark Månsson, 2008; Schwendel et al., 2015a). This might also explain the differences in monthly average FFA on ORG and CGF farms due to pasture availability and nutrient richness and diversity. In May, we observed the lowest FFA averages, and this is when fresh pasture that is rich in n-3 fatty acids is available (Mongeon, 2021). Omega-3 fatty acids are a type of UFA and are less prone to breaking down into FFA than some types of SFA (Lindmark Månsson, 2008). During later summer months (where we observed some of the highest FFA levels), farms may rely more on stored forages due to inadequate pasture availability. Stored forages have the risk of being wet and containing high levels of butyric acid, which could, therefore, contribute to the increase in FFA levels. We did not have data on the diets or frequency of diet changes, but such data would be valuable in future studies of the sources of variation in bulk milk FFA, especially on ORG and CGF farms. In addition, information on herd average days in milk and fat supplementation (for CON herds) should be considered in future studies.

In summary, the monthly average FFA from August 2018 to December 2022 was 0.83 mmol/100 g of fat with ∼7% of all monthly average FFA levels being elevated. Conventional herds had lower monthly average FFA than ORG or CGF herds. Seventy-five percent of CGF herds had at least 1 elevated monthly average FFA compared with 30% of CON herds. This research has identified a need to examine factors associated with FFA, particularly on ORG and CGF farms and to sustain milk quality. We identified yearly patterns in FFA that encourage further research, such as examining management or nutrition variables linked with periods of greater milk production demands that may be associated with FFA in CON herds.
